# Evolutionary conservation of lampbrush-like loops in drosophilids

**DOI:** 10.1186/1471-2121-8-35

**Published:** 2007-08-14

**Authors:** Roberto Piergentili

**Affiliations:** 1 Dipartimento di Genetica e Biologia Molecolare, "Sapienza" Università di Roma, Piazzale Aldo Moro 5, 00185 Rome, Italy

## Abstract

**Background:**

Loopin-1 is an abundant, male germ line specific protein of *Drosophila melanogaster*. The polyclonal antibody T53-F1 specifically recognizes Loopin-1 and enables its visualization on the Y-chromosome lampbrush-like loop named kl-3 during primary spermatocyte development, as well as on sperm tails. In order to test lampbrush-like loop evolutionary conservation, extensive phase-contrast microscopy and immunostaining with T53-F1 antibody was performed in other drosophilids scattered along their genealogical tree.

**Results:**

In the male germ line of all species tested there are cells showing giant nuclei and intranuclear structures similar to those of *Drosophila melanogaster *primary spermatocytes. Moreover, the antibody T53-F1 recognizes intranuclear structures in primary spermatocytes of all drosophilids analyzed. Interestingly, the extent and conformation of the staining pattern is species-specific. In addition, the intense staining of sperm tails in all species suggests that the terminal localization of Loopin-1 and its orthologues is conserved. A comparison of these cytological data and the data coming from the literature about sperm length, amount of sperm tail entering the egg during fertilization, shape and extent of both loops and primary spermatocyte nuclei, seems to exclude direct relationships among these parameters.

**Conclusion:**

Taken together, the data reported strongly suggest that lampbrush-like loops are a conserved feature of primary spermatocyte nuclei in many, if not all, drosophilids. Moreover, the conserved pattern of the T53-F1 immunostaining indicates that a Loopin-1-like protein is present in all the species analyzed, whose localization on lampbrush-like loops and sperm tails during spermatogenesis is evolutionary conserved.

## Background

A synthetic description of *Drosophila melanogaster *spermatogenesis comes from light microscopy studies [1-3], as well as electron microscopy studies [4-9]. At the tip of *Drosophila *testis a group of 8–9 staminal cells mitotically divide forming another staminal cell and a primary spermatogonium. Primary spermatogonia divide four times producing, after the last division, sixteen primary spermatocytes. Primary spermatocytes undergo a relatively long (~90 hours) maturation phase, during which they slowly increase their nuclear volume so that by the end of the growth phase they are 25–30 times larger than spermatogonia. Morphologically, at the beginning of their development primary spermatocytes are very similar to spermatogonia; however, the completely developed nuclei are characterized, in addition to their size, by the presence of three filamentous structures called *lampbrush-like loops*. Primary spermatocytes at late meiotic prophase I show an extensive fragmentation of these loops [10,11]. Meiosis produces 64 haploid spermatids which are easily recognizable by the association of a phase lucent nucleus with a phase dense mitochondrial derivative (*nebenkern*) of the same size (approximately 7 μm). In the last phase of spermatogenesis, spermatid nuclei reduce in volume by 200 times as a byproduct of DNA condensation and lose cytoplasmic organelles, while the nebenkern elongates and divides into two symmetric halves between which the sperm axoneme is formed. After sperm tail formation, spermatozoa transfer into seminal vesicles and they are ready to be inseminated into the female. Mature spermatozoa in drosophilids are characterized by very long sperm tails: their size is approximately 1.8 mm in *D. melanogaster*, 23 mm in *D. hydei *and almost 60 mm in *D. bifurca *[5,12-14].

The Y chromosome of *D. melanogaster *is a submetacentric, completely heterochromatic element representing 12% of the male genome [15]. The main genetic function of the Y chromosome is male fertility: X/0 flies are phenotypically normal males, but they are completely sterile [16]. It has been demonstrated that Y-associated fertility factors play a role only in the male germ line [17] and more specifically inside primary spermatocytes [5]. Three fertility factors (namely *kl-5*, *kl-3 *and *ks-1*) have huge physical dimensions [18], showing a DNA content of ~4,000 Kb each, this being 100 times longer than an average eukaryotic gene. These uncommon sizes might be partly explained by the fact that they form, inside primary spermatocyte nuclei, three giant lampbrush-like loops [10], and indeed one of the most striking features of Y-loops is their DNA content. Most of the Y chromosome DNA is represented by transposable elements [19] and simple sequence satellite DNA [20-22]. Furthermore, some of these satellites are abundantly transcribed in the ks-1 and kl-5 loops, but the corresponding transcripts do not migrate into the cytoplasm, and disintegrate together with the loops during meiotic prophase I [23]. The unusual behavior of these transcripts has lead to a debate regarding the functional role of Y-loops in drosophilids. It has been proposed that Y-loops are merely the cytological manifestation of unusually long genes (see [24] for review). Goldstein and coworkers [25] found that mutants lacking *kl-3 *and *kl-5 *loci in *D. melanogaster *do not express some high molecular weight polypeptides and lack the external dynein arms in the sperm axonemes, suggesting that these regions indeed harbor the structural genes for these proteins. In support of this hypothesis Gepner and Hays [26] found an open reading frame (ORF) coding for a dynein heavy chain inside the *kl-5 *locus (*Dhc-Yh3*); this gene is conserved also in *Drosophila hydei *in both location (Y chromosome) and cytological phenotype (a loop called Thread) [27]. Further support for this hypothesis comes from Carvalho and coworkers [28] who found another dynein heavy chain gene inside the *kl-3 *fertility factor and an occludin-related gene (*ORY*) inside the *ks-1 *locus [29]. However, although the Y loops appear to represent single giant transcription units, the presence of such extremely large introns full of repetitive DNA suggests the non-protein coding regions of the transcription units might serve other purposes. Loops can be selectively stained by Giemsa at pH10, as well as by dansyl-chloride and Coomassie blue [10]; furthermore, several antibodies have been raised which specifically recognize these structures [10,30-37], showing that they accumulate high quantities of proteins. An interesting example is provided by T53-1, a monospecific, polyclonal antibody that recognizes Loopin-1, a testis-specific autosomal protein of *Drosophila melanogaster*. Loopin-1 is present onto both the Y-chromosome kl-3 loop and the sperm tails [30]; notably, T53-1 also stains the Pseudonucleolus and, partially, the Cones Y-loops of *D. hydei*. The accumulation on the loops of proteins encoded by autosomal genes suggests that the Y-loops, besides any coding function, might also play a structural role, possibly as a nuclear framework useful for docking and/or modifying proteins to be used later in spermatogenesis [10,30,38].

In the present paper I studied the male germ lines of 13 *Drosophila *species and used the T53-F1 antibody (a second antiserum prepared against Loopin-1 which has the same specificity of the original T53-1 antiserum), to assay the evolutionary conservation of lampbrush-like loops. In all the 13 species of drosophilids analyzed, intranuclear structures were detected in primary spermatocytes using phase-contrast microscopy. Such structures resemble the Y-chromosome loops of *D. melanogaster *[5] and *D. hydei *[40], and in each species one loop is intensely stained by the antibody. The antibody shows no specific staining in spermatids but strongly decorates sperm tails. These data strongly suggest that lampbrush-like loops are a conserved, and consequently a necessary feature of drosophilid meiosis. Moreover, the conservation of spatial and temporal immunostaining in all species suggests a conserved, although unknown, function for Loopin-1. Comparison of these data with other spermatogenesis parameters, such as the size of primary spermatocyte nuclei or the sperm length, did not reveal any evident relationship, suggesting that these aspects of male germ line are not directly correlated.

## Results

### Phase-contrast analysis

**Figure 1:**
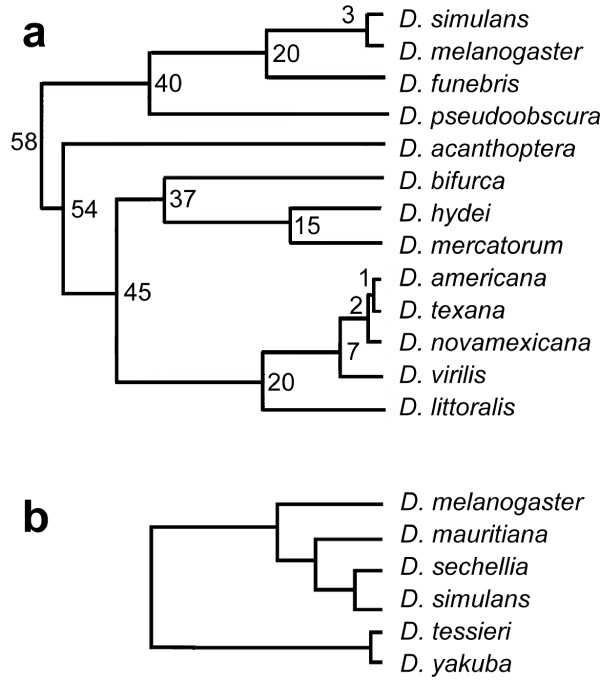
**Genealogical trees of the drosophilids analyzed in the present work**. a: general phylogeny; b: partial phylogeny of the melanogaster subgroup, an enlargement of the *simulans/melanogaster *branch of the upper tree. Numbers in panel a indicate how many million years ago the species diverged. The common ancestor of all these species is supposed to have lived some 60 million years ago; the divergence between *D. melanogaster *and *D. tessieri *probably occurred between 10 and 15 million years ago. The upper tree is partially taken and modified from [13]. The lower tree is partially taken and modified from [48].

**Figure 2:**
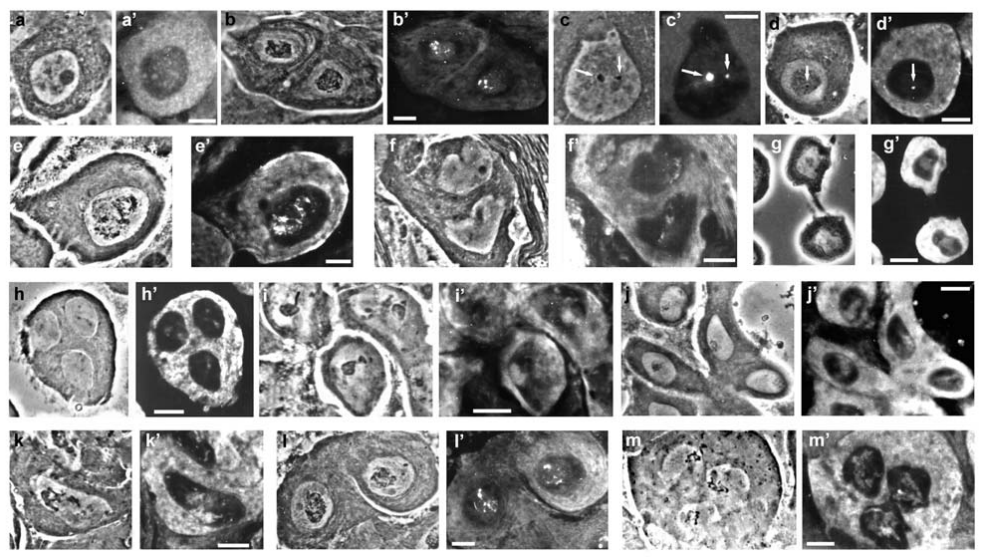
**T53-F1 immunostaining of primary spermatocyte nuclei of drosophilids**. Note that in all species it is possible to recognize intranuclear structures specifically decorated by the antibody. For all species, the first image is a phase-contrast micrograph, the second shows the corresponding immunostaining. a-a': *D. acanthoptera*. b-b': *D. americana*. c-c': *D. bifurca*. d-d': *D. funebris*. e-e': *D. littoralis*. f-f': *D. mauritiana*. g-g': *D. mercatorum*. h-h': *D. pseudoobscura*. i-i': *D. sechellia*. j-j': *D. tessieri*. k-k': *D. simulans*. l-l': *D. texana*. m-m': *D. yakuba*. Bars: 10 μm.

In order to study the evolutionary conservation of lampbrush-like loops among drosophilids (see the phylogeny in Figure 1), a detailed cytological analysis of male meiosis was carried out using phase-contrast microscopy in 13 species (Table 1). This analysis was performed using the same protocol described for *D. melanogaster *[30]. Adult or larval testes were dissected in phosphate buffer, gently squashed and immediately frozen in liquid nitrogen. After removal of the coverslip all preparations were fixed using standard methanol/acetone protocol. The results, summarized in Table 1 and illustrated in Figure 2, show that in every species primary spermatocytes could be identified based on their characteristic features, such as their large nuclear size and the presence of prominent intranuclear structures in addition to the nucleolus. Notably, these features are also typical in spermatocytes of the two well characterized species *D. melanogaster *and *D. hydei*. In many species (*D. acanthoptera, D. bifurca, D. littoralis, D. mauritiana, D. sechellia, D. tessieri, D. yakuba*) a spherical structure having a phase dense appearance is visible, which most probably corresponds to the nucleolus as it is similar in both shape and size to *D. melanogaster *or *D. hydei *nucleoli. This structure is quite different from most of the other filamentous material visible inside primary spermatocyte nuclei of all species, showing their complex cytological organization before meiosis I. A species-specific pattern for the intranuclear structures is also recognizable. In some cases it is possible to see prominent structures occupying most of the nuclear volume, as for *D. acanthoptera *(Figure 2a), *D. americana *(Figure 2b), *D. funebris *(Figure 2d), *D. littoralis *(Figure 2e), *D. sechellia *(Figure 2i), *D. simulans *(Figure 2k), and *D. texana *(Figure 2l). In other cases these structures are smaller (*D. bifurca*, Figure 2c; *D. mercatorum*, Figure 2g; *D. yakuba*, Figure 2m) or barely visible (*D. mauritiana*, Figure 2f; *D. pseudoobscura*, Figure 2h; *D. tessieri*, Figure 2j). Besides the size, also the shape of these intranuclear structures is species-specific; some of them have a filamentous aspect as seen in *D. melanogaster *loops, while other species show phase dense structures resembling some of the Y-loops of *D. hydei *(Table 1). Taken together, these data indicate that lampbrush-like structures similar to those already known in *D. melanogaster *and *D. hydei *are present in all species analyzed. It is therefore likely that these structures are present in all drosophilids, strongly suggesting a conserved role for lampbrush-like loops during spermatogenesis.

**Table 1:** Analysis of various aspects of male spermatogenesis in different drosophilids

**species (in alphabetical order)**	**sperm length (mm)**	**sperm tail entering eggs (mm)**	**T53-F1 staining in tails**	**loop size**	**Loop shape**	**overlap**	**nucleus shape**
*D. acanthoptera*	5.83 ± 0.09	(++)	yes	3	d	weak	S
*D. americana*	5.22 ± 0.02	nd	yes	1	d	weak	S
*D. bifurca*	58.29 ± 0.67	1.6	yes	1	d	strong	O
*D. funebris*	8.29	nd	yes	1	d	strong	S
*D. hydei*	23.32 ± 0.51	1.31	yes	2	d	strong	O
*D. littoralis*	7.72 ± 0.08	(++)	yes	3	f	strong	S
*D. mauritiana*	1.036	nd	yes	2	f	weak	S
*D. melanogaster*	1.9 ± 0.01	1.78	yes	3	f	strong	S
*D. mercatorum*	nd	nd	yes	3	d	strong	O
*D. novamexicana*	6.72 ± 0.15	nd	yes	3	f	weak	S
*D. pseudoobscura*	0.36	0.36	yes	2	d	strong	S
*D. sechellia*	1.649	nd	yes	2	d	weak	S
*D. simulans*	1.14 ± 0.01	1.14	yes	3	d	weak	S
*D. tessieri*	1.0 to 2.0	nd	yes	3	d	none	S
*D. texana*	5.08 ± 0.04	nd	yes	2	d	weak	S
*D. virilis*	5.70 ± 0.16	nd	yes	3	f	weak	S
*D. yakuba*	1.681	nd	yes	3	f	none	S

Data reported in the first three columns are partially taken from [13, 41, 49-51], and (++) indicates that almost the entire sperm tail enters the egg. Columns 5, 6 and 7 refer to the structures decorated by T53-F1 inside primary spermatocytes, as described in the Results section. In particular, loop size refers to the relative extension of the immunostaining inside the primary spermatocyte nucleus, 3 meaning very extensive loop, and 1 meaning very small loop. Loop shape indicates its filamentous (f) or dense (d) appearance. Overlap is "strong" when immunostaining and phase-contrast visible structures inside primary spermatocytes nuclei are the same, "weak" when the structures are only partially overlapped; "none" indicates that immunostained structures do not have a counterpart visible by phase-contrast. Nucleus shape, S indicates spherical or nearly spherical, O indicates other shapes (see Figure 2 and the Discussion section for further explanations). Cytological data about *D. melanogaster*, *D. hydei*, *D. virilis *and *D. novamexicana *partially come from [30].

### Immunostaining by T53-F1

Following the rationale stated above, testes from the same drosophilid species were fixed and immunostained with the T53-F1 antibody. Although T53-F1 was prepared against a recombinant form of Loopin-1, its specificity is the same as that of the original T53-1 antibody, which was prepared against Loopin-1 purified from *Drosophila *testes [39]. In all cases the antibody intensely decorates sperm tails (data not shown) and a specific structure inside primary spermatocyte nuclei (Figure 2). Thus a Loopin-1 related protein with a similar function appears to be present in all these species, although this indication was not confirmed by immunoblot analysis. However, clear differences exist among the species. For example in both *D. funebris *(Figure 2d') and *D. bifurca *(Figure 2c') the T53-F1 antibody stains small, dot-like loops, while in *D. mercatorum *(Figure 2g') it decorates a discoidal structure occupying the central region of the "dumbbell-shaped" primary spermatocyte nuclei. It is noteworthy that the structures recognized by the antibody do not always overlap with structures visible by phase-contrast microscopy. In some species (*D. bifurca*, *D. funebris*, *D. littoralis*, *D. mercatorum*, *D. pseudoobscura*) the overlap is evident, in other cases (*D. acanthoptera*, *D. americana*, *D. mauritiana*, *D. sechellia*, *D. simulans*, *D. texana*) it is only partial. There are also species (*D. tessieri*, *D. yakuba*) in which there is no overlap. Notwithstanding the morphological differences of the intranuclear structures recognized by T53-F1, these data clearly show that in all the 13 species tested, as well as in the other two species partially described by Pisano and coworkers [30], structures similar to *D. melanogaster *or *D. hydei *Y-chromosome lampbrush-like loops are present inside primary spermatocyte nuclei. Moreover, in all cases at least one loop is decorated by the T53-F1 antibody.

### Evaluation of other cytological parameters

In order to evaluate possible correlations between the structures revealed by T53-F1 antibody and other cytological parameters of male meiosis, other aspects (both pre- and post-meiotic) were considered. In particular, data available from the literature about sperm tail length and the amount of sperm tail entering the egg upon fertilization were considered. In addition, data about the shape of primary spermatocyte nuclei obtained in the present work or coming from the literature were collected. These parameters were compared to the relative size and shape of the lampbrush-like loops visible by immunofluorescence. It should be noted however that to date there are no data to assess if the immunostaining completely overlaps the actual size of the loops. Data used for these comparisons are summarized in Table 1. Alongside the data collected here, those of other four species (*D. melanogaster*, *D. hydei*, *D. virilis *and *D. novamexicana*) are also reported [30]. Preliminary results seem to exclude any direct correlation among these parameters.

Drosophilids are known to have sperm tails of extremely variable sizes, starting from *D. persimilis *(only 0.32 mm, similar in size to the 0.36 mm of the closely related *D. pseudoobscura *analyzed in this paper) to the giant ~60 mm sperm produced by *D. bifurca *[14]. Comparison of sperm lengths and relative loop staining extension inside primary spermatocyte nuclei reveals that these two parameters are not correlated. Indeed it is possible to find long-tailed species with a small loop (*D. bifurca*, Figure 2c-2c') as well as species with relatively short sperm tails and a very prominent loop (*D. tessieri*, Figure 2j-2j'; *D. simulans*, Figure 2k-2k'). Moreover, similarly sized sperm tails of *D. acanthoptera *(Figure 2a-2a'), *D. americana *(Figure 2b-2b') and *D. texana *(Figure 2l-2l') do not correspond to similarly sized loops.

The absence of a correlation is also evident when considering the amount of sperm tail entering the egg upon fertilization and the cytological appearance of loop structures by immunostaining. For example in *D. bifurca *only a small part of the tail enters the egg, and the loop staining appears small, but in the closely related *D. hydei*, although only a small part of the tail enters the egg, the loop *Pseudonucleolus *is much larger. Comments about the utilization by the zygote or by the females of part of sperm tails after fertilization can be found in [41] and references therein.

## Discussion

In the present study an extensive analysis of the male germ line of several *Drosophila *species was performed, in order to find conserved features. The selected species are scattered along their genealogical tree, so that it was possible to analyze even drosophilids which diverged approximately 58 million years ago (Figure 1a). Cytological analysis was performed using both phase-contrast microscopy and immunofluorescence, and the same fixing procedure was used, so it is possible to argue that the structures stained are probably evolutionary conserved. The results reported here indicate that in all species tested it is possible to recognize cells which are probably mature primary spermatocytes, as both their size and the presence of intranuclear structures are similar to both *D. melanogaster *and *D. hydei *(Figure 2). Moreover, with regard to their size and shape, the intranuclear structures are species-specific, and at least one of them is always specifically immunostained by T53-F1, a monospecific polyclonal antibody raised against the Loopin-1 protein of *D. melanogaster *[39]. In a pattern similar to that seen in *D. melanogaster*, the staining by T53-F1 is not present in the spermatids of any species, but the antibody intensely stains sperm tails from when they start developing (data not shown). This indicates that a Loopin-1-like protein is loaded onto lampbrush-like loops before meiosis and then accumulates along sperm tails in all the drosophilids analyzed.

Several cytological parameters were considered in order to find possible correlations among them, to elucidate a possible role of Loopin-1, since no mutation is known to date in the *loopin-1 *gene. However, it was not possible to find any relationship. I found that sperm length, amount of sperm tail inside the egg upon fertilization, loop staining size and loop shape are independent parameters (Table 1 and Figure 2).

Whereas Loopin-1 immunostaining was found to be conserved with regard to its presence in male germ lines, the shape and staining size of the corresponding lampbrush-like loops were found to be species-specific. In fact, although all species tested have loops stained by T53-F1 and similarly sized primary spermatocytes nuclei, all possible combinations can be found. The loop appears very small in *D. americana*, *D. bifurca *and *D. funebris *(very distant species) and it appears very prominent in *D. acanthoptera*, *D. littoralis*, *D. melanogaster *and *D. mercatorum *(similarly distant species) (Figure 1). However, it seems that the shape of primary spermatocyte nuclei may be conserved. The only three species having a non spherical nucleus are *D. bifurca*, *D. hydei *and *D. mercatorum*, which are evolutionary close to each other (Figure 1a). The first two show a "pear-like" nucleus, while the last species has an "dumbbell-shaped" nucleus (Figure 2 and Table 1). Notably, all three species have loops of a dense nature, suggesting a similar molecular organization. In this respect it is interesting to note that a filamentous loop recognized by T53-F1 is present only in some species of the melanogaster subgroup (Figure 1B), and in the closely related species *D. novamexicana*, *D. virilis *and *D. littoralis *(Table 1). The conservation of nuclei shape, staining size and appearance is even more interesting if we compare it with previous reports [42,43]. The sperm tail length and sperm head size are very variable not only among species, but also among different populations of the same species and even inside the same individual [44]. This suggests that mechanisms controlling premeiotic stages are more stringent than those controlling sperm development.

Thus far the biological role of Loopin-1 in *Drosophila melanogaster *male germ line is unknown. The *loopin-1 *gene maps to polytene region 53C [39] and its product was originally described as a tektin-like protein [30]. Subsequent studies, however, demonstrated that the *loopin-1 *gene corresponds to the CG4750 locus of *D. melanogaster *and is homologous to a leucine aminopeptidase [39,45]; therefore, it is unrelated to the tektin family of proteins. The protein encoded by this gene is conserved at least in *D. simulans *(55% identities, 72% positives) which diverged from *D. melanogaster *3My ago, and in *D. pseudoobscura *(78% identities, 88% positives) which diverged from *D. melanogaster *40My ago (Figure 1). This is not surprising, since it has been recently demonstrated that leucine aminopeptidases represent an important fraction of the *Drosophila *sperm proteome [49], although their role in sperm tail is still unclear. It is also known that Loopin-1 localization in *D. melanogaster *sperm tails is not dependent upon loop binding [30]. Indeed, its pattern of localization is quite peculiar: the protein recognized by the T53-F1 antibody appears inside primary spermatocyte nuclei during their maturation, binding to the kl-3 loop throughout its development [30]. At the beginning of meiosis, as soon as Y-chromosome lampbrush-like loops disgregate, staining fades and no other sign of the protein is present during meiosis or spermatid maturation. The staining once again becomes intense in sperm tails at the time they begin to elongate. Notably, this peculiar behavior was found to be similar in all the 13 species examined, despite their considerable phylogenetic distance (Figure 1). The fact that T53-F1 reacts specifically in a conserved pattern strongly suggests that *D. melanogaster *Loopin-1 is a conserved protein and that the formation of loop-like structures during primary spermatocyte development is a necessary step during spermatogenesis in all species examined.

## Conclusion

The data reported here show that a Loopin-1-like protein is detectable in both primary spermatocyte nuclei and sperm tails in all species tested, and possibly in all drosophilids. These data strongly suggest that this protein might have an important functional role during spermatogenesis through its binding to intranuclear structures during the development of primary spermatocyte nuclei. Notably, the structures decorated by the T53-F1 antibody during primary spermatocyte nuclei development clearly resemble the well known Y-chromosome lampbrush-like loops of *D. melanogaster *and *D. hydei*. It is likely that lampbrush-like loops might be a sign of Y chromosome activity during spermatogenesis in all drosophilids tested. Therefore it would not be surprising to find that the Y chromosome plays a similar role in the development of lampbrush-like loops in all drosophilids.

Evolutionary data demonstrate that drosophilids diverged at least 60 million years ago. Data shown here support the hypothesis that a Loopin-1-like protein exists in all species tested, indicating that it is evolutionary conserved. Moreover, data presented here also demonstrate that primary spermatocyte nuclei of all species tested show the presence of discrete intranuclear structures, indicating that this cytological feature is also conserved. The lack of a *loopin-1 *mutation however has impeded the assessment of its role during spermatogenesis, and the function of Loopin-1 loading onto lampbrush-like loops in the male germ line. Interestingly, mutations in other genes that, like *loopin-1*, are located on the autosomes have recently been shown to affect the formation of the kl-3 loop and to alter the external dynein arms of the sperm axonemes [47] which are believed to be the site of action of the dynein encoded by the *kl-3 *fertility factor. The spermatozoa of these mutants either degenerate before maturation or have major structural defects leading to their complete immobility. These data suggest that these autosomal proteins might be involved in building the structure of the lampbrush-like loops – either to set up structures necessary for transcription of the *kl-3 *gene or to perform other functions associated with the loop.

## Methods

### Strains

*D. bifurca*, *D. acanthoptera *and *D. littoralis *were kindly provided by the National Drosophila Species Resource Center at Bowling Green State University (OH – USA); all the other species are part of the collection of *Drosophila *stocks of Prof. M. Gatti and Prof. S. Bonaccorsi at the University of Rome "Sapienza". All lines were reared at 25°C using standard cornmeal medium.

### Cytology and immunostaining

Larvae or adults of all species were dissected and testes were fixed and analyzed as described in [30], with the exception of very long testes, as in *D. bifurca*. In this case 24 × 32 mm coverslips were used, and testes were gently unfolded with forceps before squashing. This protocol preserves the intranuclear structures visible during *in vivo *analysis, as demonstrated for *D. melanogaster *and *D. hydei*. The T53-F1 antibody was kindly provided by Dr. Pisano. The T53-1 antibody was obtained injecting *Drosophila melanogaster *testis proteinaceous extracts into mice. Subsequently the *loopin-1 *gene was identified (CG4750) using the T53-1 antibody to isolate *E. coli *clones expressing the protein. This recombinant protein was again injected into mice and the T53-F1 antibody was obtained. The complete procedure for the isolation of bacterial clones and the characterization of the T53-F1 staining pattern by both immunofluorescence and western blot analyses were extensively described in the PhD Thesis of Dr. Gambino [39]. Since there is no exhaustive description of male germ lines of the species used in the present work, it was assumed that the largest cells observed inside testes, which also showed intranuclear structures besides the nucleolus, were indeed mature primary spermatocytes, as is the case for *D. melanogaster *and *D. hydei*.

## Authors' contributions

RP designed the study, carried out all experiments and prepared the manuscript.
